# Telemedicine for Pregnant Women on a Japanese Remote Island: A Two-year Report

**DOI:** 10.31662/jmaj.2022-0195

**Published:** 2023-09-27

**Authors:** Yuta Ishikawa, Kentaro Nakanishi, Akio Masuda, Misa Hayasaka, Ai Tsumura, Koji Murakami, Takeshi Umazume, Tetsuzou Masuda, Kunihiko Nishiwaki, Yasuhito Kato

**Affiliations:** 1 Department of Obstetrics and Gynecology, Wakkanai City Hospital, Hokkaido, Japan; 2 Department of Obstetrics and Gynecology, Asahikawa Medical University, Hokkaido, Japan; 3 Rebun Town National Health Insurance Funadomari Clinic, Hokkaido, Japan; 4 Department of Obstetrics and Gynecology, Hokkaido University Graduate School of Medicine, Hokkaido, Japan

**Keywords:** antenatal care, maternal health service, pregnancy, telemedicine, ultrasonography

## Abstract

**Introduction:**

Remote antenatal checkups were conducted on the northernmost island of Japan to reduce the burden of hospital visits among pregnant women. This study aims to investigate the effectiveness and safety of remote antenatal checkups for pregnant women living on a remote island.

**Methods:**

This observational study included singleton pregnancies on Rebun Island between October 2020 and September 2022. General surgeons conducted medical interviews and performed fetal sonography using an obstetrician videoconference system at the main central hospital. The primary outcomes were the degrees of physical, mental, and economic burdens of hospital visits and the levels of anxiety and satisfaction with remote antenatal checkups as assessed using a questionnaire survey. Moreover, we investigated the incidence of adverse perinatal events, including maternal death, fetal death, neonatal death, severe neonatal neurological disorders, and other obstetric complications.

**Results:**

This study included 16 out of 22 pregnant women from Rebun Island who visited the central hospital. No adverse perinatal events occurred as a result of the remote antenatal checkups. One pregnant woman had gestational diabetes, whereas the others had no obstetric complications. The participants underwent a median of two remote antenatal checkups. According to a questionnaire survey, 90.0%, 80.0%, and 70.0% of the pregnant women perceived improvements in their physical, mental, and economic burdens, respectively. Although 70.0% of the participants experienced anxiety regarding remote antenatal checkups before the introduction, all were satisfied after delivery.

**Conclusions:**

Remote antenatal checkups effectively reduced the burden of hospital visits for pregnant women, who reported high levels of satisfaction. Furthermore, antenatal checkups were safely conducted on remote islands.

## Introduction

In Japan, owing to the chronic shortage of obstetricians and gynecologists, the consolidation of delivery facilities has been progressing to efficiently use limited medical resources. However, this consolidation creates disparities in accessibility to antenatal care among regions ^(1)^. Most pregnant women undergo approximately 14 antenatal checkups in Japan. However, hospital visits can be physically, mentally, and economically cumbersome for pregnant women living in depopulated areas.

Telemedicine is one of the most promising approaches to address this problem. Several studies have reported that its introduction as a part of antenatal care has reduced the number of hospital visits without compromising safety because of the coronavirus disease 2019 (COVID-19) pandemic ^(2), (3), (4)^. One systematic review reported that telemedicine improved obstetric outcomes and optimized hospital visit schedules for pregnant women at high risk ^(5)^. Other studies have reported that a telemedicine system, in which fetal sonography that is performed by a nonspecialist in a low-resource area is evaluated by specialists, has improved diagnostic performance ^(6), (7)^ and reduced the time and treatment costs for pregnant women and their families ^(8)^. However, despite the reported promising results, integrating telemedicine into daily practice is challenging ^(9), (10)^ as it requires policy support, patient demands, and collaboration between facilities and professionals. In Japan, a system to introduce telemedicine for antenatal checkups is absent. Furthermore, issues regarding the introduction and maintenance of telemedicine systems, such as cost, manpower, and responsibility, have hindered their widespread use.

Wakkanai City Hospital is the only facility that handles births in the 4,600 km^2^ Soya region of Japan, which includes two remote islands, one of which, Rebun Island, has a population of 2,400 and is located 60 km west of Wakkanai City on the northernmost island of Hokkaido in the Sea of Japan. The only hospital on the island, the Rebun Town National Health Insurance Funadomari Clinic, has two full-time general surgeons. No onsite obstetricians or gynecologists are available, and only one visiting obstetrician-gynecologist from Wakkanai City Hospital visits once a month to provide obstetric and gynecologic care. Therefore, pregnant women living on the island have to spend approximately 3 h, including a 2-h boat ride, to visit Wakkanai City Hospital.

We conducted a social implementation study on remote medical checkups for pregnant women living on Rebun Island using a cloud-based medical video communication system to reduce the burden of antenatal hospital visits. Two years have passed since the introduction of remote antenatal checkups, and we investigated the impact of remote antenatal checkups on the burden on pregnant women. In addition, we evaluated the safety of this system for pregnant women living on remote islands.

## Materials and Methods

The remote antenatal checkup initiative on Rebun Island has started on October 1, 2020. This observational study included pregnant women who resided on Rebun Island and visited Wakkanai City Hospital between October 1, 2020, and September 30, 2022. This study was approved by the Institutional Review Board of Wakkanai City Hospital (R3-5). Written informed consent was obtained from women who lived on Rebun Island during their first prenatal visit. Women with multiple pregnancies, those with maternal medical complications requiring treatment, and those who did not provide consent for remote antenatal checkups were excluded from the study. The base hospital was established at Rebun Town National Health Insurance Funadomari Clinic, and the central hospital was located at Wakkanai City Hospital. Pregnant women from Rebun Island who visited Wakkanai City Hospital received an explanation for the study design at an outpatient clinic. Pregnant women who provided written informed consent to participate in this study underwent antenatal checkups at Rebun Town National Health Insurance Funadomari Clinic.

Pregnant women with a gestational age of 15-34 weeks were eligible for remote antenatal checkups. They underwent screening for placental abnormalities at approximately 20 weeks of gestation, fetal screening at approximately 30 weeks of gestation by obstetrician-gynecologists, and antenatal guidance by midwives after 34 weeks of gestation at the central hospital. A single fetal screening scan was performed at 30 weeks of gestation to determine the appropriate delivery location―either the central hospital or a more advanced perinatal facility. Fetal screening was performed according to the guidelines of the Japan Society of Obstetrics and Gynecology. Pregnant women at ≥37 weeks of gestation were admitted to the hospital for delivery. Pregnant women could receive antenatal checkups and medical care at the central hospital at any time that they desired. An anonymous questionnaire was administered after delivery to evaluate the effectiveness of remote antenatal checkups. Table 1 presents the contents of the questionnaires.

**Table 1.** Remote Antenatal Checkup Self-Administered Questionnaire Evaluation for Postpartum Women.

**1-1**	Gravida
**1-2**	Parity
**1-3**	Working during pregnancy
**2-1**	Complications during pregnancy
**2-2**	How many times did you need to make an unscheduled visit to the central hospital?
**3-1**	Did you feel any physical burden of the hospital visits?
**3-2**	Did you feel any mental burden of the hospital visits?
**3-3**	Did you feel any economic burden of the hospital visits?
**4-1**	Did you have any anxiety about undergoing a remote prenatal checkup?
**4-2**	Were you satisfied with your remote prenatal checkups?
**4-3**	Has telemedicine improved your physical burden?
**4-4**	Has telemedicine improved your mental burden?
**4-5**	Has telemedicine improved your economic burden?

The remote antenatal checkup comprised medical interviews, weight measurements, blood pressure measurements, urinalyses, and fetal sonographies. Blood tests were performed at approximately 24 weeks of gestation to check for anemia and glucose levels. Pregnant women were prescribed medications for pregnancy-related symptoms. Physicians at both hospitals connected to the videoconferencing system on their laptops at the time of the scheduled examinations of pregnant women. For real-time video communication, we used Kizuna Web (Borderless Vision Corp., Sapporo, Japan), a cloud-based video communication platform that uses a transport layer system for encrypted communication over ordinary internet lines. The remote antenatal checkup and fetal ultrasonography were performed by a general surgeon at the base hospital, and an obstetrician-gynecologist at the central hospital confirmed the checkup in real time. Pregnant women suspected of obstetrical complications such as fetal growth restriction, placental abnormalities, or threatened premature labor received subsequent checkups at the central hospital until these complications were ruled out. Pregnant women at risk of preterm birth less than 36 weeks or massive postpartum hemorrhage such as placenta previa were transferred to a more advanced perinatal facility.

The primary outcomes were the degrees of physical, mental, and economic burdens of hospital visits and the degrees of anxiety and satisfaction with remote antenatal checkups assessed using a questionnaire survey. The secondary outcome was the incidence of perinatal adverse events. Adverse perinatal events were defined as maternal death, fetal death, neonatal death, or serious neurological disorders in neonates. In addition, we investigated the incidence of other obstetrical complications, including hypertensive disorders of pregnancy, gestational diabetes, fetal growth restriction, placental abnormalities, threatened premature labor, pregnancy outcomes (vaginal delivery, cesarean delivery, and transfer to another facility), and the number of remote antenatal checkups. All data were analyzed using descriptive statistics.

## Results

A total of 22 pregnant women from Rebun Island visited the central hospital during the study period, of which 16 pregnant women were enrolled in the study. Six pregnant women were excluded for several reasons. Two were excluded because of scheduling conflicts, whereas the remaining four were excluded for suspected placenta previa, gestational diabetes, threatened miscarriage, or unknown reasons. All patients were included in this study during the early stages of pregnancy. Table 2 presents the maternal and neonatal characteristics. The median age of the participants was 29 years (range, 23-36 years). Six patients (37.5%) were nulliparous. Complications during pregnancy were observed in only one patient (6.3%) with gestational diabetes. Remote antenatal checkups were employed in 21.1% (27/128) of all antenatal checkups, with a median of 2 (range, 1-3). Approximately 43.8% (7/16) of pregnant women were eventually referred to medical facilities in their parents’ hometown for delivery (Table 2). None of the participants required transfer to other medical facilities. No adverse perinatal events occurred as a result of the remote prenatal checkups.

**Table 2.** Maternal and Neonatal Characteristics (n = 16).

Characteristics	Values
Maternal age (years)	30 (23-36)
Nulliparous	6 (37.5)
Frequency of telemedicine (times)	2 (1-3)
1 time/woman	7 (43.8)
2 times/woman	8 (50.0)
3 times/woman	1 (6.2)
Obstetrical complication	1 (6.2)
Gestational diabetes	1 (6.2)
Delivery at the central hospital	9 (56.3)
Gestational week of delivery (weeks)^a^	38.5 (38-40)
Vaginal delivery^a^	7 (77.8)
Birth weight (g)^a^	3,153 (2,510-3,594)
Male neonate^a^	5 (55.5)
Adverse perinatal events^a^	0

Values are presented as median (range) or n (%). ^a^Six out of seven pregnant women transferred to other perinatal facilities were excluded from this analysis because their perinatal outcomes were unknown.

**Figure 1.**
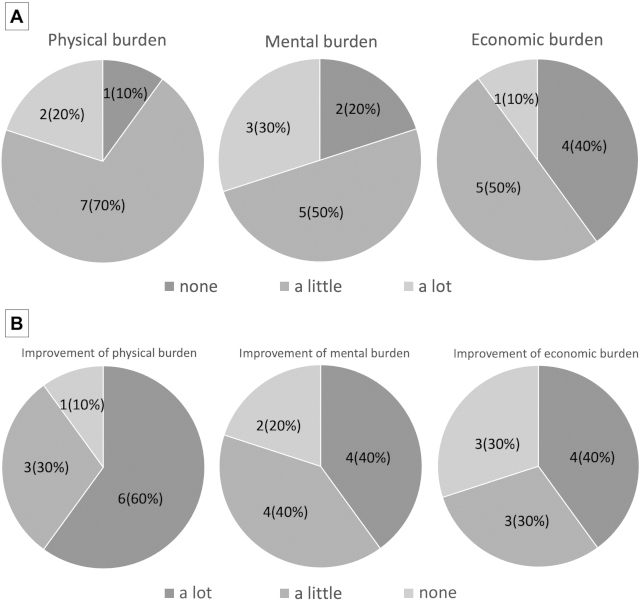
A. Self-assessment of the physical, mental, and economic burdens of hospital visits on pregnant women before the introduction of remote antenatal checkups. B. Self-assessment of the physical, mental, and economic burdens of hospital visits on pregnant women after delivery.

**Figure 2.**
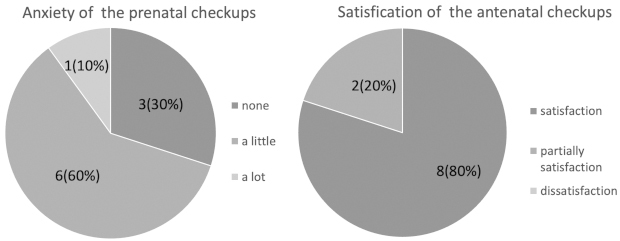
Self-assessment of participants’ anxiety and satisfaction regarding remote antenatal checkups.

The response rate of the postpartum questionnaire was 62.5% (10/16). Overall, 90.0% (9/10), 80.0% (8/10), and 60.0% (6/10) of pregnant women experienced physical, mental, and economic burdens of hospital visits, respectively (Figure 1A). After delivery, 90.0% (9/10), 80.0% (8/10), and 70.0% (7/10) of pregnant women stated that telemedicine reduced their physical, mental, and financial burdens, respectively (Figure 1B). Most participants had little concern regarding remote antenatal checkups, and none was dissatisfied (Figure 2).

## Discussion

In this study, we reported that remote antenatal checkups could help in reducing the burden of hospital visits for pregnant women living on remote islands. In addition, remote antenatal checkups can be safely performed on a remote island using a videoconferencing system, with obstetricians supporting nonobstetricians.

The COVID-19 pandemic has increased the demand for telemedicine to reduce hospital visits. Replacing certain components of antenatal care, such as medical interviews, lifestyle guidance, and counseling, with telemedicine can improve access to obstetric care without significant adverse events ^(3)^. In addition, the diagnostic performance of fetal sonography by nonspecialists in low-resource areas was reportedly improved by telemedicine evaluation by perinatologists ^(6), (7)^. Moreover, the present study reported that obstetric care for low-risk pregnancies can be safely provided on remote islands with limited medical resources. In addition, special devices using cloud-based communication systems are not required. Few studies have assessed the costs of implementing and maintaining remote obstetric care. This system may be implemented at a lower cost than that of using a mobile antepartum cardiotocogram ^(4)^, a dedicated tablet, or a personal computer to share fetal sonography videos ^(6)^. A survey on telemedicine in Japan reported that the additional cost that pregnant women could pay for remote antenatal checkups was <10 USD ^(11)^.

In this study, all participants were sufficiently or partially satisfied with the remote antenatal checkups and no negative opinions were received regarding telemedicine. Jagannathan et al. assessed the findings of a self-administered questionnaire and reported that 86.9% of pregnant women who received antenatal telemedicine were satisfied with the care that they received ^(3)^. However, in a survey conducted in Japan on remote antenatal checkups, only approximately 30% of pregnant women responded that they were satisfied or more satisfied with remote checkups than with face-to-face care ^(11)^. In this previous study, the omission of fetal sonography in telemedicine affected the satisfaction with telemedicine (rather than the fact that it was not face-to-face care). In Japan, fetal sonography is customarily performed at every antenatal checkup visit and pregnant women look forward to seeing their fetuses during ultrasonographic examinations. Therefore, participants’ satisfaction with the remote antenatal checkup might have been higher in this study because fetal sonography was performed by a physician at the base hospital. Remote antenatal checkups have helped in reducing the burden of hospital visits for pregnant women living on Rebun Island. However, telemedicine did not ease the economic burden, probably because the local government fully subsidizes transportation and accommodation costs for face-to-face antenatal checkups.

This study is the first report of remote antenatal checkups, including fetal sonography, performed by general surgeons on a remote island in Japan. However, it had several limitations. First, seven pregnant women (43.8%) were transferred to other hospitals during the study period, which might have affected the results. Second, the lack of adverse perinatal events might be attributed to the small number of participants. Therefore, further research is required to validate our findings and obtain a clearer understanding of the benefits of telemedicine. Third, the number of remote antenatal checkup visits conducted was low. Pregnant women living on Rebun Island had a maximum of five opportunities to undergo remote antenatal checkups. However, the median number of telemedicine visits per pregnant woman was two. Even if we exclude the seven pregnant women who were transferred to other facilities during the follow-up period, the median number of telemedicine visits remained relatively small. The low frequency of telemedicine use might be one contributing factor because opportunities for telemedicine are available only once a month. It was not uncommon for remote antenatal checkup dates to not match the participants’ schedules. Other reasons for the low frequency of telemedicine use were that obstetrician-gynecologists went on business trips once a month to provide normal antenatal checkups, and pregnant women in Rebun after 37-week gestation stayed in Wakkanai for delivery. We are planning to increase the number of remote antenatal checkups. Finally, the present study was conducted during the COVID-19 pandemic, which might have had a positive impact on the participants’ evaluations in this study because many people did not go out to avoid infection.

In conclusion, pregnant women living on a remote island in Japan were able to reduce the burden of hospital visits without the adverse perinatal events associated with remote antenatal checkups. As the demand for telemedicine is expected to increase in Japan, research on remote antenatal checkups requires continuation and the remote checkup option should be validated for its effectiveness and safety.

## Article Information

### Conflicts of Interest

None

### Sources of Funding

This work was supported by the [Department of the Center of Innovation, Hokkaido University] JST COI grant number: [JPMJCE1301].

### Acknowledgement

We would like to express our gratitude to Masanori Yoshino (Center for Intellectual Property and Innovation, Hokkaido University, Sapporo, Japan) and Borderless Vision Corp. (Sapporo, Japan) for their involvement in this telemedicine project.

We would also like to thank Editage (www.editage.com) and Sharon Hanley (Department of Women’s Health Medicine, Hokkaido University, Sapporo, Japan) for English language editing.

### Author Contributions

Yuta Ishikawa: conception and design, acquisition of data, analysis and interpretation of data, drafting of the manuscript, and statistical analysis.

Kentaro Nakanishi: conception and design, critical revision of the manuscript for important intellectual content, and supervision.

Akio Masuda: acquisition of data.

Misa Hayasaka: acquisition of data and analysis and interpretation of data.

Ai Tsumura: acquisition of data.

Koji Nakamura: acquisition of data.

Takeshi Umazume: supervision.

Tetsuzou Masuda: supervision.

Kunihiko Nishiwaki: acquisition of data.

Yasuhito Kato: supervision.

### Approval by Institutional Review Board (IRB)

R3-5, Wakkanai City Hospital, Hokkaido, Japan
